# The Effect of Workplace Pressure and Experience on Burnout in Embryologists Working in Assisted Reproduction in Spain

**DOI:** 10.3390/ijerph23030369

**Published:** 2026-03-13

**Authors:** Raquel Urteaga, Amelia Díaz

**Affiliations:** Faculty of Psychology, University of Valencia, Avd Blasco Ibañez, 21, 46010 Valencia, Spain; raurgar@alumni.uv.es

**Keywords:** embryologists, assisted reproduction, burnout, experience, workplace pressure

## Abstract

**Highlights:**

**Public health relevance—How does this work relate to a public health issue?**

- Approximately 12% of births in Spain are the result of Assisted Reproduction (AR).
- The work performed by embryologists working in AR is of vital importance in the successful process of AR, consequently, their workplace wellbeing is a public health issue.

**Public health significance—Why is this work of significance to public health?**

- Spanish embryologists working in AR show a burnout pattern with moderate to high levels of emotional exhaustion, depersonalization, and low personal accomplishment.
- The causal pattern found in the burnout dimensions is emotional exhaustion as the first and close dimension to workplace pressure, affecting both depersonalization and low personal accomplishment.

**Public health implications—What are the key implications or messages for practitioners, policy makers and/or researchers in public health?**

- Interventions in the embryologist’s workplace are necessary to promote their own physical and mental health.
- Burnout prevention protocols are necessary due to potential negative consequences that burnout in embryologists could have in the AR process.

**Abstract:**

Professionals working in Assisted Reproduction (AR) have shown high levels of burnout, with embryologists being the most affected. Previous studies have found that burnout shows symptomatology when professionals are exposed to long-lasting workplace stressors. Therefore, the main objectives of this study were estimating burnout levels in embryologists working in AR and testing whether the observed associations between the variables measured fit existing theoretical models. A cross-sectional design was used in a sample of 127 Spanish embryologists working in AR. Years working in AR, excessive workplace pressure and burnout dimensions, emotional exhaustion, depersonalization, and lack of personal accomplishment were measured. Results showed that burnout was present in significant percentages of embryologists working in AR (35.4%, 42.5%, and 28.3% showing high levels of emotional exhaustion, depersonalization, and lack of personal accomplishment, respectively). Additionally, relational and mediational analyses provided support for the Leiter and Maslach’s theoretical model where emotional exhaustion was the first and closer variable to the stressor high pressure in the workplace, followed by depersonalization and lack of personal accomplishment. Implications arising from this study directly affect the mental health of embryologists and their performance on the job, where interventions targeting perceived workplace pressure may reduce burnout indicators in embryologists working in AR.

## 1. Introduction

Assisted Reproduction (AR) is a subsection in the gynecology and obstetrics medical branches dedicated to helping women facing difficulties conceiving naturally to become pregnant; more specifically “reproduction brought about through ovulation induction, controlled ovarian stimulation, ovulation triggering, AR procedures, and intrauterine, intracervical, and intravaginal insemination with semen of husband/partner or donor” (pp. 2686) [1]. Professionals working in AR vary from the usual gynecologists and nurses to professionals such as embryologists, psychologists, nurse assistants, andrologists, geneticists, nutritionists, and lab technicians.

The literature in the field has shown that professionals working in AR present high levels of burnout, with embryologists being the most affected [2,3,4,5,6]. Two main studies have been performed to explain the burnout in these professionals, the Integrated Approach to Fertility Care study by Boivin et al. [7], focused on the day-to-day involvement with patients as the major source of burnout, and Fanchim et al.’s study [8], where attention has been given to three main sources of burnout: the communication between AR professionals and patients in the first place; the organizational–personality interaction, where environment stressors are integrated by the professionals according to their personality traits, coping styles, and work skills in second place; and the last one, which is closely associated with the responsibility of the work performed, where success was measured by achieving the desired goal, a pregnancy, whilst non-success was the loss of or failure to obtain a pregnancy. Different studies have confirmed these sources of stress in AR professionals, pointing towards aspects such as working under pressure, overloaded schedule, lack of professional recognition, communication difficulties between the team members, management of complicated patients, and breaking bad news to patients [2,9,10,11,12,13,14,15].

In a previous work [2], the authors focused their efforts on the study of the two sources of burnout proposed by Boivin et al. [7] and Fanchim et al. [8]: communication between AR professionals and patients as the first source, where the main stressors found were communication difficulties between the team members, management of complicated patients, breaking bad news to patients, and working under excessive pressure as the second source, including overloaded schedule and lack of professional recognition. The above-mentioned work revealed that the stressor that specifically affected the higher percentages of embryologists was working under excessive pressure. This stressor in embryologists has been associated with high responsibility in the success in AR fecundation, the high workload, performing long, tedious, and repetitive tasks in the lab and the heavy paperwork [4,5,6,16,17,18]. This pattern is compatible with the assumption proposed by Maslach [19] that burnout shows symptomatology when professionals are exposed to long-lasting stressors. Additionally, a longitudinal study in healthcare employees found that if the long-lasting stressors are maintained in the workplace, year after year, the burnout levels will increase accordingly [20]. Therefore, it can be assumed that embryologists working under the effect of long-lasting stressors will increase their burnout levels as they accumulate years of experience in that type of work. This picture shows the necessity to study burnout in these professionals and find out the role of variables such as excessive pressure in the workplace and time working in these conditions.

Burnout as a psychological phenomenon was first described in the United States, linked to research conducted in the mid-1970s to explain the decline in the quality of care and professional attention provided to service users. It has been described as a psychological syndrome that occurs in response to chronic and long-lasting stressors on the job [21]. Nowadays, there seems to be a consensus that burnout is a three-dimensional syndrome characterized by emotional exhaustion, depersonalization, and lack of personal accomplishment in the workplace. Maslach [19] described these three dimensions originally related to professionals working in a service workplace. Emotional exhaustion, considered to be the closest to a stress variable, was characterized by feelings of depletion and exhaustion of emotional resources, as a feeling of having nothing left to offer others. Depersonalization was described as having feelings of emotional hardening, detachment, alienation, loss of the ability to connect, and the adoption of negative, cynical, cold, and distant attitudes toward service recipients. Finally, the lack of personal fulfillment referred to the appearance of negative feelings of inadequacy and lack of competence and professional effectiveness, with decreased personal expectations, which implies a negative self-evaluation where rejection of oneself and a feeling of lack of personal achievements can develop, as well as feelings of failure and low self-esteem. Thus, burnout syndrome is characterized by emotional exhaustion that could lead to depersonalization and low personal accomplishment in a gradual progressive process.

However, the relationship among burnout dimensions is a controversial issue [22]. According to the Leiter and Maslach’s multidimensional model [23], the long-lasting exposure to stressors triggers the central and more obvious symptom, emotional exhaustion, which represents the basic individual stress dimension of burnout. Nevertheless, emotional exhaustion misses a crucial point, the relationship professionals have with their job, more associated with depersonalization and lack of personal accomplishment dimensions. When exhaustion is present, depersonalization arises as a defensive, cognitive-emotional coping strategy, where professionals create distance between themselves and service recipients by viewing them as objects, rather than individuals, displaying cynical attitudes especially when they are exhausted. Finally, lack of personal accomplishment or feelings of inefficacy and low self-evaluation could appear when emotional exhaustion, depersonalization, or both, are present. Summarizing, for Leiter and Maslach, emotional exhaustion is the first and central dimension from which the other two dimensions are derived [23]. Alternative models propose different directional relationships between dimensions as shown in Table 1. Golembiewski et al. highlighted depersonalization as the first dimension [24], whereas Lee and Ashforth’s model proposed emotional exhaustion as the initial dimension, tightly related to depersonalization, whereas lack personal accomplishment would be independent from depersonalization [25,26].

The last study on burnout focusing specifically on embryologists in Spain, as far as we know, was published in 2014 [5], twelve years ago, whilst more recent data from AR professionals including a small subsample of embryologists, was published in 2024 [2]. Results in this more recent study clearly showed higher burnout levels in embryologists than those reported in the 2014 study, hence the importance of having more complete and up-to-date information on burnout levels. Consequently, the aims of this study were to estimate burnout levels in embryologists working in AR and test whether the observed associations between the variables fit existing theoretical models. According to these aims, the hypotheses proposed were as follows:

1. Significant percentages of embryologists would show burnout in the dimensions of emotional exhaustion, depersonalization, and lack of personal accomplishment.
2. Relationships between years working in AR, workplace pressure and burnout dimensions, emotional exhaustion, depersonalization, and lack of personal accomplishment would be significant and positive.
3. Emotional exhaustion and depersonalization would play a serial mediating role between the stressor years working in AR and lack of personal accomplishment.
4. Emotional exhaustion and depersonalization would play a serial mediating role between the stressor excessive workplace pressure and lack of personal accomplishment.

## 2. Materials and Methods

### 2.1. Participants, Design, and Procedure

The sample is composed of 127 embryologists from both, the Spanish Association for the Study of Reproductive Biology (ASEBIR) and the Spanish Society of Fertility (SEF), representing 21.2% of the 600 embryologists in these societies. The survey was conducted online for two months, from 8 July to 8 September 2024. The age mean was 39.35 years (SD = 8.86 years) and the age-range was 24–64. According to gender, 101 (79.5%) were women and 26 (20.5%) were men. The study used a cross-sectional design. Permission to carry out the research was obtained from ASEBIR, SEF, and the Ethical Committee for Scientific Research of the authors University (protocol code 3468621/27 October 2023). The informed consent included a description of the study and a link that gave access to it.

### 2.2. Measures

The Spanish version of the Maslach Burnout Inventory (MBI-HSS) [27] validated in different Spanish professionals [28] was completed by the participants. This inventory is composed of 22 items with a response Likert scale (0 = never, 1 = a few times a year or less, 2 = once a month or less, 3 = a few times a month, 4 = once a week, 5 = a few times a week, to 6 = every day), according to the frequency that the described statement happens. The inventory assesses burnout through the subscales of emotional exhaustion, depersonalization, and personal accomplishment. The personal accomplishment items have been reversed to assess lack of personal accomplishment. Specifically, higher scores in emotional exhaustion, depersonalization, and lack of personal accomplishment indicate high levels of burnout. The reliability obtained through Cronbach’s α for the MBI-HSS in this study was adequate for the three subscales: 0.81 for emotional exhaustion, 0.73 for depersonalization, and 0.72 for lack of personal accomplishment. There are two methods to interpret burnout from this inventory, both used in our study: the sums of the items in each subscale and the distributions of respondents in the three levels of low, moderate, and high (see Table 2) using the following cut-points [29]:

The survey included questions about demographic variables such as gender, age, and two related to their jobs, years working in AR, and excessive pressure supported in the workplace.

### 2.3. Statistical Analysis

Descriptive statistics are presented in the first place, including means, standard deviations, kurtosis, and skewness, the last two values to assess the normal distribution of the burnout scores, years working in AR and excessive workplace pressure. Secondly, gender differences were calculated for all variables in the study. In third place, the percentages of embryologists in each category of burnout were obtained, presenting the three levels of low, moderate, and high for each of the subscales of burnout. In fourth place, the relationships between these subscales, years working in AR, and excessive workplace pressure were calculated. Finally, in trying to find out the compatibility of the data with theoretical models, exploratory multiple serial mediation analyses were performed to test which burnout dimension plays a more significant role, as mediator or alternatively as dependent variable, in all cases having years working in AR or, alternatively, excessive workplace pressure, as independent variables. Statistical analyses were conducted with SPSS 28.0 (IBM Corporation, Armonk, NY, USA) software and Hayes [30] PROCESS macro for SPSS.

## 3. Results

### 3.1. Descriptive Statistics

The means and standard deviations of burnout subscales are presented in Table 3. The time embryologists had been working in AR ranged from 1 year to 37 years, showing a mean of 12.98 years (SD = 8.86, Skewness = 0.73, Kurtosis = −0.08). Regarding excessive workplace pressure, 23 (18.1%) and 104 (81.9%) answered No and Yes, respectively, to the question “Do you support excessive pressure at your workplace?”. No variable included in the study presented skewness and kurtosis values higher than 2 or lower than −2 [31] being in all cases close to zero [32], assuming normal distribution in these variables. Results show that burnout was present at a high level in the subscales of depersonalization, with 42.5%, and emotional exhaustion, with 35.4%, of embryologists. Lack of personal accomplishment was less representative, with 28.3%, of these professionals.

No significant gender differences were found in the study’s variables: emotional exhaustion t (125) = −0.48, *p* = 0.634; depersonalization t(125) = 1.03, *p* = 0.304; lack of personal accomplishment t(125) = −0.31, *p* = 0.764; years working in AR t(125) = −0.07, *p* = 0.947 and excessive pressure at the workplace t(125) = −1.61, *p* = 0.117.

**Figure 1:**
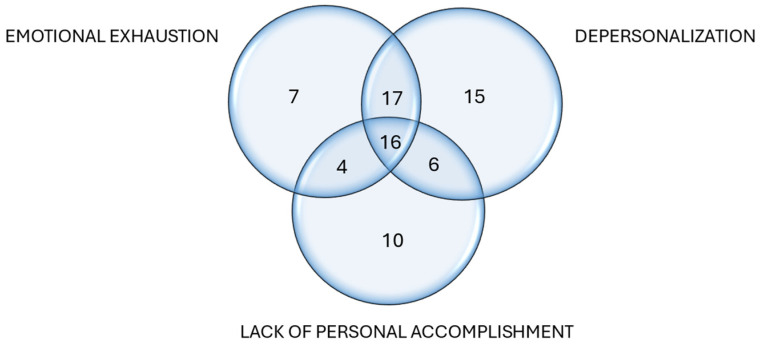
Venn diagram of the number of embryologists with high levels of emotional exhaustion, depersonalization, and/or lack of personal accomplishment.

Figure 1 presents data of the 75 (59%) embryologists with high emotional exhaustion, depersonalization, and/or lack of personal accomplishment, the main symptoms of burnout, and the overlap between them. The remaining 52 (41%) embryologists showed moderate and low signs of burnout. Additionally, Figure 1 shows that 16 (21.33%) embryologists presented high levels in the three dimensions of burnout, 33 (44%) showed high emotional exhaustion and depersonalization, six (8%) reported high depersonalization and lack of personal accomplishment, and four (5.33%) informed of high emotional exhaustion and lack of personal accomplishment. Therefore, the pattern of burnout presented by embryologists is characterized mainly by emotional exhaustion and depersonalization.

### 3.2. Relationships

Table 4 presents the relationship between the three dimensions of burnout and the variables years working in AR and excessive workplace pressure. Years working in AR showed positive relations with emotional exhaustion and depersonalization, but negative with lack of personal accomplishment, significant with depersonalization and lack of personal accomplishment and non-significant with emotional exhaustion. No relationships were shown between years working in AR and excessive workplace pressure. On the other hand, excessive workplace pressure presented significant and positive relationships with emotional exhaustion, depersonalization and lack of personal accomplishment. The relationships between the three burnout dimensions presented a typical pattern with positive and highly significant relationships between them.

Finally, based on the relationships presented by the study’s variables, two different exploratory multiple serial mediation analyses were performed based on ordinary least squared regression and the bootstrap method. The indirect effects were obtained with 95% bias-corrected bootstrap confidence interval on 10,000 bootstraps. When the variable years working in AR was the independent one in the mediational analysis, and the burnout dimensions took alternatively the mediation or dependent variable role, controlling for the covariates gender and age, no mediation analysis produced a significant indirect effect.

**Figure 2:**
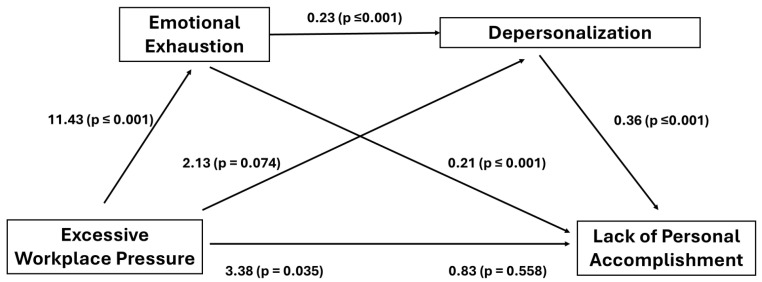
Serial mediation of emotional exhaustion and depersonalization between workplace pressure and lack of personal accomplishment. Unstandardized B values.

Nevertheless, when excessive workplace pressure was the independent variable with emotional exhaustion and depersonalization as serial mediators, lack of personal accomplishment as dependent variable, and gender and age as covariates, the exploratory multiple serial mediation was significant, producing a full mediation (see Figure 2). The direct effect of (1) of excessive workplace pressure on emotional exhaustion (B = 11.43, SE = 2.56, t = 4.47, *p* ≤ 0.001, 95% CI [6.36, 16.50]) was statistically significant; (2) the direct effect of emotional exhaustion on depersonalization was significant (B = 0.23, SE = 0.39, t = 6.03, *p* ≤ 0.001, 95% CI [0.16, 0.31]); (3) the mediation of emotional exhaustion between excessive workplace pressure and depersonalization was not significant (B = 2.13, SE = 1.18, t = 1.80, *p* = 0.074, 95% CI [−0.22, 4.46]). In addition, (4) the direct effect of depersonalization on lack of personal accomplishment was significant (B = 0.36, SE = 0.11, t = 3.36, *p* ≤ 0.001, 95% CI [0.15, 0.57]); (5) the indirect effect of emotional exhaustion on lack of personal accomplishment through depersonalization (B = 0.21, SE = 0.52, t = 4.00, *p* ≤ 0.001, 95% CI [0.11, 0.31]) was also significant. Finally, (6) the direct effect of excessive workplace pressure on lack personal accomplishment was significant (B = 3.28, SE = 1.54, t = 2.13, *p* = 0.035, 95% CI [0.23, 6.34]), significance that was lost when emotional exhaustion and depersonalization were introduced to the equation (B = −0.83, SE = 1.42, t = −0.59, *p* = 0.558, 95% CI [−3.65, 1.98]). The overall model was significant (F(3123) = 13.37, *p* < 0.001) and explained 35.6% of the total variance. The covariates gender and age did not produce any statistically significant effects in the exploratory serial mediation analysis (B = 0.86, SE = 1.26, t = 0.68, *p* = 0.495, 95% CI [−1.63, 3.35]; B = −0.10, SE = 0.06, t = −1.68, *p* = 0.096, 95% CI [−2.21, 0.02] for gender and age respectively).

Table 5 presents the three significant indirect effects, (1) the mediation effect of emotional exhaustion between excessive workplace pressure and lack of personal accomplishment, (2) the mediation effect of depersonalization between excessive workplace pressure and lack of personal accomplishment, and, finally, (3) the indirect effect of the serial mediation of emotional exhaustion and depersonalization between excessive workplace pressure and lack of personal accomplishment.

## 4. Discussion

One of the main aims of the present work was to update the burnout data regarding Spanish embryologists, since the most recent data from a study specifically performed on embryologists was published in 2014 [5], and higher burnout levels were detected compared to those of 2014, in a recent study from 2024 in a subsample of embryologists analyzed with other professionals working in AR [2]. Our study, focusing exclusively on embryologists, shows a burnout pattern where emotional exhaustion and depersonalization are the main protagonists. Even in the lack of personal accomplishment burnout subscale, the least representative of embryologists in our sample, almost 3 out of 10 showed lack of personal accomplishment. Therefore, with more than half of the sample of embryologists showing signs in at least one of the three burnout dimensions, and 2 out of 10 presenting the three of them, the first hypothesis about the high level of burnout in embryologists working in AR is confirmed in the three dimensions assessed, depersonalization, emotional exhaustion and lack of personal accomplishment. Although the percentages of embryologists showing burnout symptoms in this study are notable, they are much lower than those obtained in the study performed in 2024 [2], analyzing all professionals working in AR (72%) where specifically, the percentages of embryologists in the highest level were 72.1%, 48.1% and 48.1% for emotional exhaustion, depersonalization and lack of personal accomplishment respectively. These high levels of burnout in embryologist are consistent with previous studies [2,3,4,5,6,9,10,11,12,13,14,15], placing them below the levels found in UK and USA [6] for emotional exhaustion, 59% and 62% respectively, but at similar level in depersonalization to those found in UK (42%), although lower that in USA (60%) [6]. Therefore, according to our results, a significant percentage of embryologists in our sample are characterized by feelings of detachment and indifference towards their work and people, such as colleagues, clients or patients, accompanied by feelings and sensations of tiredness, fatigue, and lack of emotional energy to cope with work tasks. Our results show a profile where depersonalization and emotional exhaustion are predominant, with lack of personal accomplishment less representative of our sample, where embryologists scoring higher emotional exhaustion or depersonalization did not always report higher lack of personal accomplishment. This pattern was also found in nurses in Turkey, with depersonalization being the most prominent dimension in that study, and lack of personal accomplishment the less prominent [33].

Two theories arise as possible explanations of the burnout pattern found in this study, the Social Exchange Theory and the Organizational Theory. The Social Exchange Theory [34] proposed that burnout is based on the inequality between effort put into doing the work and the results obtained from it. These efforts are emotionally consuming whereas the results are not recognized, with this being the original source of emotional exhaustion and depersonalization as a stress coping strategy. The most relevant result of our study, the high percentages of embryologists presenting depersonalization and emotional exhaustion in our sample, perfectly matches the core of this theory. Additionally, it must be noticed that one of the main complaints from embryologists is the lack of professional recognition [35]. In the other hand, the Organizational Theory emphasizes the organizational and work stressors as the sources of burnout, accompanied by inadequate individual coping strategies [36,37]. Among the organizational and work stressors in embryologists there are several that stand out, these include: overloaded schedules, work under excessive pressure, communication difficulties between the team members, management of complicated patients, breaking bad news to patients, low salary, isolation, poor ergonomics, and lack of natural light in the laboratories [2,35]. The effect of these stressors could create in the workers a decrease in their organizational commitment, accompanied by high levels of emotional exhaustion and depersonalization and cynicism as a coping strategy. Again, this theory would adequately explain our results, since burnout in our sample was characterized by high levels of depersonalization and emotional exhaustion.

The second hypothesis proposed regarding the relationships between the embryologists’ experience, measured as years working, workplace pressure and the three dimensions of burnout were partially confirmed. The relationships between the burnout dimensions both between them and with the excessive workplace pressure variable are consistent with the expected results. This last stressor, as an organizational one has positive and significant relationships with the three burnout dimensions, showing in all cases positive and significant effects, supporting previous findings [16,17,18]. However, the relational role of experience in the job, measured as years working in AR did not entirely match the expected results, showing no relation with excessive workplace pressure, negative relation with lack of personal accomplishment, but positive relation with emotional exhaustion and depersonalization. Working for years in a stressful environment, where excessive workplace pressure is present, seems to be related with burnout mainly in the form of emotional exhaustion and depersonalization, but, at the same time, these years of experience seem to be consistent with a positive effect on lack of personal accomplishment, where a significant percentage of embryologists perceived themselves as having professional efficacy, competence, and productivity at work, experiencing a growing sense of adequacy about their ability to perform the job well, and presenting a good self-evaluation of their job. The type of work performed by embryologists, usually in labs, working with cells [38], and following strict protocols could explain why some of these professionals do not feel that burnout affects their self-perceived efficacy or competence.

Regarding the relationships among the three burnout dimensions, and more specifically, the mediator or dependent variable role that they could play when the independent variable was, alternatively, years working in AR or excessive workplace pressure, results from the exploratory mediation analyses showed that years working, as an independent variable, did not induce any mediational effect on any of the burnout dimensions, independently of the role they play, as mediator or dependent variable, giving no support to hypothesis three. It seems that day-to-day stressors could be more related to burnout dimensions than to the number of years working. Similar results were found in the field of caregivers of a dependent person, where the number of years being a caregiver showed no relation with perceived burden or stress, but it was the hours per day dedicated to caregiving or the care tasks performed that accounted for the caregivers’ perceived burden and mental health symptoms [39]. In long-term situations, when looking at the effect of time, researchers always must consider the adaptation and coping processes humans undertake [40].

On the other hand, excessive workplace pressure seems to be closely related to burnout dimensions, supporting previous studies [16,17,18]. Exploratory serial mediational analysis, based on multiple regressions, having emotional exhaustion and depersonalization as serial mediators, gave rise to a significant indirect effect between the stressor workplace pressure and lack of personal accomplishment, resulting in a full mediation. Our results support the basic role of emotional exhaustion, but also the role of depersonalization, where both variables, directly and indirectly, are linked significantly to a lack of personal accomplishment, giving support to the fourth hypothesis. From the three theoretical models proposed [23,24,25,26], our data does not support the Golembiewski et al. [24] model since, while an important stressor, as workplace pressure operated, it is the emotional exhaustion dimension that is more directly and strongly associated, instead of depersonalization. The Lee and Ashforth’s [25,26] model is consistent with our results only partially; emotional exhaustion is the basic and closer dimension to workplace pressure, but since the mediation of emotional exhaustion between workplace pressure and depersonalization was not significant, it seems that the more independent variable is depersonalization instead of lack of personal accomplishment as Lee and Ashforth [25,26] proposed. Finally, our data seems to be more consistent with the model proposed by Leiter and Maslach [23], where emotional exhaustion is the first and basic dimension, that could be followed by depersonalization as a defensive coping strategy, and lack of personal accomplishment with feelings of inefficacy and low self-evaluation. Other studies looking at the association pattern in the burnout dimension found depersonalization for men and emotional exhaustion for women as the first dimensions in GPs [41]. Following these findings, and considering that our sample was made up by an almost 80% of women, our results are aligned with those found in GPs. Finally, the study performed by Önder and Basim [33] analyzing both burnout levels and association pattern in burnout dimensions in nurses, found a burnout dimensions profile similar to ours, with emotional exhaustion and depersonalization at the highest levels, showing lack of personal accomplishment at the lowest.

In the context of Spain, since the first official records in 2014 [42], there has been a steady increase in the percentages of births resulting from AR techniques, estimated at 33% up to 2021 [43], despite the negative effect of the COVID-19 pandemic on health services in general, with the last official records available indicating that around 12% of births, almost 40,000 in 2022, were the result of AR techniques [44]. This increase in demand for AR services may be the root cause of the observed burnout in AR professionals in general and more specifically in embryologists. The few studies, to our knowledge, performed on these professionals showed a picture where burnout is present at high levels, mainly in the dimensions of emotional exhaustion and depersonalization, a fact that has important implications in both their own mental health and in the negative consequences that any error in their work could cause. Our study updates and confirms these results and, as a direct implication, highlights the need to improve the work environment of embryologists working in AR to decrease the pressure they perceive. Increasing the number of embryologists to reduce individual workload and improving time organization, among other strategies could improve the perception of workplace pressure. But looking at other studies, there are more stressors in the embryologist workplace than just the excessive pressure. Other common stressors are overloaded schedules, lack of professional recognition, communication difficulties between the team members, management of complicated patients, and breaking bad news to patients [2,9,10,11,12,13,14,15]. Personality traits that can directly influence the perception of these stressors [8], such as neuroticism, which could be a potential vulnerability factor, cannot be ignored either [45].

The present work has shed light on Spanish embryologists with respect to burnout; however, burnout in embryologists from other counties, having different work conditions could present different pattern to the one found here. Among other limitations of the present study is the fact that cross-sectional designs, like the one used in our work, are unable to make inferences about causal relationships. Also, the sample is small and of convenience with unbalanced gender participants; consequently, the size of the sample could reduce the reliability of the results obtained from the statistical analyses performed. Future research should include a higher number of embryologists and a balanced gender sample that would make it possible to perform gender-stratified analyses. Additionally, all information obtained came from self-reports, therefore the study’s results may be influenced by the limitation associated with the assessment tool used, such as social desirability in the case of self-reports. The exploratory nature of the variable excessive workplace pressure, assessed as a dichotomous variable, does not rely on a validated scale and excessively reduces its variability, it would be advisable to use a Likert-type scale or a validated occupational stress measure in future studies. Finally, although gender and age have been controlled in the exploratory mediational analysis, other variables not controlled could potentially play a confounding role in the results of the study.

Future research in the field should consider the limitations just mentioned and put into practice what Basar called “the balance model” [46] balancing the human and the technological elements in the embryologist’s work. Sometimes, because of the advancement of assisted reproduction techniques and the cutting-edge technology used in the laboratories, the human factor is overlooked. A balanced approach would prioritize empathy, fluent communication between staff members and consequently also with patients, emotional intelligence training and promotion of well-being in the work environment. General interventions to reduce emotional exhaustion and depersonalization include stress management/adaptative coping programs, assertiveness training, reducing the workload, increasing human resources, psychosocial intervention [47,48], and more specifically for embryologist organizational programs to reduce excessive workplace pressure, sharing responsibilities, having a work schedule where weekend and vacations are respected, working hours in the labs combined by rest periods, and increasing the contact with patients could have a positive impact on a better mental health and wellbeing in this professionals.

## 5. Conclusions

The present study found a burnout pattern in Spanish embryologists characterized by high levels of emotional exhaustion and depersonalization. Lack of personal accomplishment is the burnout dimension less affected in the sample studied. The results suggest an indirect association between workplace pressure and lack of personal accomplishment through emotional exhaustion and depersonalization dimensions. Additionally, the results obtained are consistent with the Leiter and Maslach [23] model regarding the dimension of emotional exhaustion as the first and basic burnout dimension that could be followed by depersonalization and lack of personal accomplishment. The most important implication arising from this study is that burnout directly affects the mental health of embryologists and may have negative consequences for the vital outcomes of their work. A comprehensive study of organizational, emotional, physical, and job-specific stressors should be conducted to isolate, eliminate, or at least reduce their effect on the work of embryologists. Similarly, interventions focused on the well-being of these professionals would positively impact their mental health and reduce the risk of negative outcomes in their work.

## Figures and Tables

**Table 1:** Relationship pattern between the burnout dimensions from the three models.

Models	Burnout Dimensions
Leiter and Maslach	Emotional Exhaustion → Depersonalization → Lack of Personal Accomplishment
Golembiewski, Munzenrider and Stevenson	Depersonalization → Lack of Personal Accomplishment → Emotional Exhaustion
Lee and Ashforth	Emotional ↗ Depersonalization Exhaustion ↘ Lack of Personal Accomplishment

**Table 2:** Cut points in the burnout dimensions categories.

	Low	Moderate	High
Emotional Exhaustion	≤18	19–26	≥27
Depersonalization	≤5	6–9	≥10
Lack of Personal Accomplishment	≥40	34–39	≤33

**Table 3:** Skewness, kurtosis, means, standard deviations, categorization (low, moderate and high levels) of Maslach Burnout Inventory subscales in the whole sample.

	Skewness	Kurtosis	Mean (SD)	Low n (%)	Moderate n (%)	High n (%)
Emotional exhaustion	0.47	−0.83	23.63 (11.67)	55 (43.3)	27 (21.3)	45 (35.4)
Depersonalization	0.34	−0.78	9.23 (5.76)	36 (28.3)	37 (29.1)	54 (42.5)
Lack of personal accomplishment	0.55	−0.11	11.24 (6.80)	48 (37.8)	43 (33.9)	36 (28.3)

SD = Standard deviation.

**Table 4:** Relationships between years working in AR, excessive workplace pressure, and burnout dimensions.

	1	2	3	4	5
1. Years working in AR	___				
2. Excessive workplace pressure	−0.04	___			
3. Emotional exhaustion	0.15	0.38 ***	___		
4. Depersonalization	0.25 **	0.30 ***	0.52 ***	___	
5. Lack of personal accomplishment	−0.23 **	0.19 *	0.51 ***	0.51 ***	___

* = *p* ≤ 0.05; ** = *p* ≤ 0.01; *** = *p* ≤ 0.001.

**Table 5:** Indirect effects of emotional exhaustion and depersonalization and between excessive workplace pressure and lack of personal accomplishment.

Effects	Point Estimate	SE	R^2^	Bootstrapping 95% Confidence Interval	
				Lower	Upper
1. EWP → EE →LPA	2.39	0.64	0.28	1.19	3.68
2. EWP → D → LPA	0.77	0.38	0.27	0.12	1.59
3. EWP → EE → D → LPA	0.96	0.34	0.35	0.39	1.73

EWP = Excessive Workplace Pressure; EE = Emotional Exhaustion; D = Depersonalization; LPA = Lack of Personal Accomplishment. SE = Standard error; R^2^ = Variance explained by the models.

## Data Availability

The datasets generated and analyzed during the current study are available from the corresponding author on reasonable request. Data available on request due to restriction: Privacy (we did not request approval for the release of the individual participants data in our study).

## Abbreviation

The following abbreviation is used in this manuscript:

AR	Assisted Reproduction

## References

[1] Zegers-Hochschild F., Adamson G., de Mouzon J., Ishihara O., Mansour R., Nygren K., Sullivan E., van der Poe S. (2009). On behalf of ICMART and WHO. The International Committee for Monitoring Assisted Reproductive Technology (ICMART) and the World Health Organization (WHO) Revised Glossary on ART Terminology, 2009. Hum. Reprod..

[2] Urteaga R., Díaz A. (2024). Burnout in assisted reproduction professionals: The influence of stressors in the workplace. Healthcare.

[3] Kasraie J., Kennedy H. (2024). Best practice for embryology staffing in HFEA licensed assisted conception centers-guidance from Association of Reproductive & Clinical Scientists. Hum. Fertil..

[4] Priddle H., Pickup S., Hayes C. (2021). Occupational health issues experienced by UK embryologists: Informing improvements in clinical reproductive science practice. Hum. Fertil..

[5] López-Lería B., Jimena P., Clavero A., Gonzalvo M.C., Carrillo S., Serrano M., López-Regalado M.L., Olvera C., Martínez L., Castilla J.A. (2014). Embryologists’ health: A nationwide online questionnaire. J. Assist. Reprod. Genet..

[6] Murphy A., Lapczynski M.S., Proctor G., Glynn T.R., Domar A.D., Gameiro S., Palmer G.A., Collins M.G. (2024). Comparison of embryologist stress, somatization, and burnout reported by embryologists working in UK HFEA-licensed ART/IVF clinics and USA ART/IVF clinics. Hum. Reprod..

[7] Boivin J., Bunting L., Koert E., Ieng U.C., Verhaak C. (2017). Perceived challenges of working in a fertility clinic: A qualitative analysis of work stressors and difficulties working with patients. Hum. Reprod..

[8] Facchin F., Leone D., Tamanza G., Costa M., Sulpizio P., Canzi E., Vegn E. (2020). Working with infertile couples seeking assisted reproduction: An interpretative phenomenological study with infertility care providers. Front. Psychol..

[9] Rausch D.T., Braverman A.M. (2020). Burnout rates among reproductive endocrinology nurses: The role of personality and infertility attitudes. Fertil. Steril..

[10] Kilstein O.S.G. (2022). The Review of the Experience of Assisted Reproductive Technology (ART) Nurse When Their Patients’ Treatment Results in a Failed Attempt to Conceive. Ph.D. Thesis.

[11] Khoa L.D., Quang T.N., Vinh D.Q., Anh N.T.N., Tuong H.M., Foster K. (2018). The prevalence of job stressors among nurses in private in vitro fertilization (IVF) centers. Nurs. Open.

[12] Moradi Y., Baradaran H.R., Yazdandoost M., Atrak S., Kashanian M. (2015). Prevalence of burnout in residents of obstetrics and gynecology: A systematic review and meta-analysis. Med. J. Islam. Repub. Iran..

[13] Thorsen V.C., Tharp A.L., Meguid T. (2011). High rates of burnout among maternal health staff at a referral hospital in Malawi: A cross-sectional study. BMC Nurs..

[14] Govardhan L.M., Pinelli V., Schnatz P.F. (2012). Burnout, depression and job satisfaction in obstetrics and gynecology residents. Conn. Med..

[15] Di Trani M., Spoletini R., Renzi A., Monaco S., Fedele F., Scaravelli G. (2024). The cultural representations and symbolizations emerging from Italian psychologists working in multidisciplinary assisted reproduction teams: A linguistic analysis with the emotional text mining. J. Health Psychol..

[16] Collins M.G., Venier W., Salhia A., Beltsos A., Lee J.A., Copperman A.B., Bailey J., Sakkas D., Broussard A. (2022). Working with fatigue: Assessment of cryomanagement conditions in IVF biorepositories. Fertil. Steril..

[17] Dolcos S.M., Daley D. (2009). Work pressure, workplace social resources, and work–family conflict: The tale of two sectors. Int. J. Stress. Manag..

[18] Murphy A., Lapczynski M., Proctor G., Meyer E.C., Glynn T., Domar A., Gameiro S., Palmer G., Collins M. (2023). The occupational challenges reported by UK embryologists: Stress, fatigue, and burnout. Hum. Reprod..

[19] Maslach C. (2003). Job Burnout: New directions in research and intervention. Curr. Dir. Psychol. Sci..

[20] Maslach C., Leiter M.P., Fink G. (2017). Burnout. Handbook of Stress Series Volume 1, Stress: Concepts, Cognition, Emotion, and Behavior.

[21] Maslach C., Schaufeli W.B., Leiter M.P. (2001). Job burnout. Annu. Rev. Psychol..

[22] Taris T.W., Leblanc P.M., Schaufeli W.B., Schreurs P.J.G. (2005). Are there causal relationships between the dimensions of the Maslach Burnout Inventory? A review and two longitudinal tests. Work. Stress..

[23] Leiter M.P., Maslach C. (1988). The impact of interpersonal environment on burnout and organizational commitment. J. Organ. Behav..

[24] Golembiewski R.T., Munzenrider R.F., Stevenson J.G. (1986). Phases of Burnout: Developments in Concepts and Applications.

[25] Lee R.T., Ashforth B.E. (1993). A longitudinal study of burnout among supervisors and managers: Comparisons between the Leiter and Maslach (1988) and Golembiewski et al. (1986) models. Organ. Behav. Hum. Decis. Process.

[26] Lee R.T., Ashforth B.E. (1996). A meta-analytic examination of the correlates of the three dimensions of job burnout. J. Appl. Psychol..

[27] Maslach C., Jackson S.E., Zalaquett C.P., Wood R.J. (1997). Maslach Burnout Inventory. Manual. Evaluating Stress: A Book of Resources.

[28] Gil-Monte P.R. (2005). Factorial validity of the Maslach Burnout Inventory (MBI-HSS) among Spanish professionals. Rev. Saúde Pública.

[29] Maslach C., Jackson S.E., Leiter M.P. (1986). Maslach Burnout Inventory.

[30] Hayes A.F. (2013). Introduction to Mediation, Moderation, and Conditional Process Analysis: A Regression-Based Approach.

[31] George D., Mallery P. (2010). SPSS for Windows Step by Step: A Simple Guide and Reference 17.0 Update.

[32] Hair J.F., Hult G.T.M., Ringle C.M., Sarstedt M.A. (2022). A Primer on Partial Least Squares Structural Equation Modeling (PLS-SEM).

[33] Önder Ç., Basim N. (2008). Examination of developmental models of occupational burnout using burnout profiles of nurses. J. Adv. Nurs..

[34] Schaufeli W.B., Maassen G.H., Bakker A.B., Sixma H.J. (2011). Stability and change in burnout: A 10-year follow-up study among primary care physicians. J. Occup. Organ. Psychol..

[35] Astro G., Gatti M., Costa M., Cosmelli A., Alteri A., Anastai A., Cimadomo D., de Santis L., Klinger F.G., Licata E. (2025). Perceived workplace stressors and professional experiences of clinical embryologists working in Italy and Spain: A pilot qualitative study. Front. Public. Health.

[36] Cox T., Kuk G., Leiter M., Schaufeli W.B., Maslach C., Marek T. (1993). Burnout, health, work stress and organizational healthiness. Professional Burnout: Recent Developments in Theory and Research.

[37] Golembiewski R.T., Munzenrider R., Carter D. (1983). Phases of progressive burnout and their work site covariants: Critical issues in OD research and praxis. J. Appl. Behav. Sci..

[38] Go K.J. (2015). By the work, one knows the workman: The practice and profession of the embryologist and its translation to quality in the embryology laboratory. Reprod. Biomed. Online.

[39] Díaz A., Ponsoda J.M., Beleña A. (2020). Optimism as a key to improving mental health in family caregivers of people living with Alzheimer’s disease. Aging Ment. Health.

[40] Cameron H.A., Schoenfeld T.J. (2018). Behavioral and structural adaptations to stress. Front. Neuroendocrinol..

[41] Houkes I., Winants Y., Twellaar M., Verdonk P. (2011). Development of burnout over time and the causal order of the three dimensions of burnout among male and female GPs. A three-wave panel study. BMC Public. Health.

[42] Health Ministry. Spanish Government (2020). Los Tratamientos de Reproducción Asistida en España Aumentan un 28% en los Últimos 5 años [Assisted Reproductions Treatments Increased by 28% in the Last 5 Years in Spain]. https://www.sanidad.gob.es/gabinete/notasPrensa.do?id=5067.

[43] Spanish Society of Fertility (SEF) (2024). Aumenta un 33% los Nacimientos por Reproducción Asistida en España, Según el Registro de Actividad de 2021 [Births Through Assisted Reproduction in Spain Increased by 33%, According to the 2021’s Activity Register]. https://sefertilidad.net/index.php?seccion=blog&subSeccion=detalleBlog&id=O9prsUk_Y20sr1GCV7wHxCRmBAQXIxjjXNICegOsXXg&title=Aumenta+un+33%25+los+nacimientos+por+reproducci%C3%B3n+asistida+en+Espa%C3%B1a%2C+seg%C3%BAn+el+registro+de+actividad+de+2021.

[44] Spanish Society of Fertility (SEF) (2024). Registro Nacional de Actividad 2022 [National Activity Registry 2022]. https://sefertilidad.net/documentos/QrOPUTXSABq8_Y3Sf1X55jeBA9MZUk_KBKRIMfq5TH8.pdf.

[45] Roloff J., Kirstges J., Grund S., Klusmann U. (2022). How strongly is personality associated with burnout among teachers? A Meta-analysis. Educ. Psychol. Rev..

[46] Basar M. (2024). Enhancing outcomes in IVF laboratories: Navigating the human element through leadership and emotional intelligence. J. Assist. Reprod. Genet..

[47] Bes I., Shoman Y., Al-Gobari M., Rousson V., Guseva Canu I. (2023). Organizational interventions and occupational burnout: A meta-analysis with focus on exhaustion. Int. Arch. Occup. Environ. Health.

[48] Ruotsalainen J., Serra C., Marine A., Verbeek J. (2008). Systematic review of interventions for reducing occupational stress in health care workers. Scand. J. Work. Environ. Health.

